# Factors Affecting the Attractive Force of Dental Magnetic Attachment: A Literature Review for Guiding Dentists in Clinical Application

**DOI:** 10.1155/2022/9711285

**Published:** 2022-06-14

**Authors:** An-Nissa Kusumadewi, Lisda Damayanti, Rukiah, Risdiana

**Affiliations:** ^1^Biotechnology Doctoral Program, Universitas Padjadjaran, Jl. Dipati Ukur No. 35, Bandung, West Java 40132, Indonesia; ^2^Departement of Prosthodontics, Faculty of Dentistry, Universitas Padjadjaran, Jl. Raya Bandung-Sumedang Km 21, Jatinangor, Sumedang, West Java 45363, Indonesia; ^3^Department of Chemistry, Faculty of Mathematics and Natural Sciences, Universitas Padjadjaran, Jl. Raya Bandung-Sumedang Km 21, Jatinangor, Sumedang, West Java 45363, Indonesia; ^4^Department of Physics, Faculty of Mathematics and Natural Sciences, Universitas Padjadjaran, Jl. Raya Bandung-Sumedang Km 21, Jatinangor, Sumedang, West Java 45363, Indonesia

## Abstract

**Aim:**

The aim of this review is to get a comprehensive description of the factors that may influence the attractive force of the dental magnetic attachment.

**Background:**

Dental magnetic attachment is a term for a magnet used as an overdenture retainer. Magnets that are widely used are permanent magnets such as neodymium iron boron (NdFeB) and samarium cobalt (SmCo). Theoretically, the magnetic attractive force in a permanent magnet has a constant retentive force, and the magnitude of the force will not decrease over time. However, several studies revealed that the magnetic attractive force can be decreased, resulting in the failure of overdenture retention. Some of the factors of reduced magnetic attraction that have been studied are corrosion and temperature. There are no articles that specifically review the factors that can influence magnetic attraction. Review Results. A total of 25,880 articles were obtained during a search on 3 journal databases: PubMed (2,647), ScienceDirect (23,184), and Scopus (229). From those publications, 15 articles reported relevant outcome data that were then extracted. Magnetic attractive force can be influenced by temperature, corrosion, keeper surface morphology, type of magnet, keeper-assembly size combination, inclination, insertion-removal cycle, gliding/loading cycle, number of magnets, crosshead speed, and force direction.

**Conclusion:**

Many factors can affect the magnetic attraction force of the dental magnetic attachment. Corrosion is the most likely factor to occur because the dental magnetic attachment is always in the oral environment which contains corrosive saliva and is susceptible to damage due to mastication forces.

## 1. Introduction

Magnet is a metal alloy that can attract certain metals such as iron, cobalt, nickel, and other alloys. It is also capable of producing a pull or repulsion of other magnets [1]^.^ Magnets are generally divided into two groups, soft magnets and strong/hard magnets. Soft magnets are magnets that are easy to magnetize and demagnetize, whereas strong/hard magnets are materials that are difficult to magnetize and demagnetize, which are often referred to as permanent magnets [2, 3]. Permanent magnets have been used in prosthodontics. Magnets in prosthodontics are used as a retainer in maxillofacial prostheses and overdenture. Magnets as an overdenture retainer are known as dental magnetic attachments [4].

Dental magnetic attachment is a retention system for dentures consisting of a magnetic assembly and a keeper. Magnetic assembly is the main part of the dental magnetic attachment, and this part contains magnet that is attached to the denture base, while the keeper is a metal part that is attached to the tooth root [4]. The dental magnetic attachment was first introduced in 1941 and was used as a retainer for mandibular dentures in cases of severe mandibular resorption [5]. The use of dental magnetic attachments before 1990 had several constraints including large magnetic size, magnetic field leakage, low retentive force, and corrosion of magnets [6], so dental magnetic attachments were rarely used for these reasons.

Various attempts were made to improve the function of dental magnetic attachments. The type of magnet widely used recently as a dental magnetic attachment is permanent magnets of rare earth magnets [2, 3] such as samarium cobalt, neodymium-iron-boron, and samarium iron nitride [3]. The advantage of using permanent magnets is that in a smaller size, it has a sufficient attractive force for a denture retention system [7]. Magnet encapsulation using stainless steel or titanium can protect magnets from direct contact with the oral environment so that they are resistant to corrosion [7].

Theoretically, the magnetic attractive force of a permanent magnet has a constant retentive force, and the magnitude of the force will not decrease over time [4, 8]. Evidently, the magnetic force can be reduced if under extreme conditions including high temperature, large external magnetic field, or changes caused to the chemical composition of the magnet due to corrosion and physical damage of the magnet [4]. The protective casing of magnets still also has the risk of corrosion or damage. Corrosion or damage to the protective casing can cause magnetic damage; as a result, the magnetic attracting force is decreased [9, 10]. Several studies reported factors that can affect the magnetic attractive force such as corrosion, temperature, and air gap [4, 9, 10]. Here, we reported a systematic review of factors that may influence the attractive force of the dental magnetic attachment to get a comprehensive description that can be useful for guiding dentists in clinical applications.

## 2. Materials and Methods

### 2.1. Search Strategy

A systematic literature search was conducted using PubMed, ScienceDirect, and Scopus in accordance with the PRISMA (Preferred Reporting Items for Systematic Reviews and Meta-Analyses) guidelines. Electronic search was conducted using the keyword combination: “Magnetic attachment OR magnetic overdenture OR dental magnetic AND attractive force OR magnetic force.”

To get more relevant articles, the search in the database is then continued by adding additional filters in the form of year limits from 2004 to 2022, research articles in English, full text article available, subjects were limited in dentistry, medicine, and materials science.

### 2.2. Screening and Selection

The inclusion criteria of the selected papers were in vitro studies that conduct research on the factors affected on the attractive force of the dental magnetic attachment. Searching in the database, screening, and selection of papers were carried out by two authors (Kusumadewi and Risdiana). The first step is screening the title of the articles. If the title is irrelevant, then the article is removed. If the title is relevant, then the abstract is read carefully. Abstracts were analyzed whether they fit the inclusion criteria and eligible for review. If there was any doubt, the full text of the article was read. Dubious papers are then discussed with all of the authors to decide whether to be removed or included in the review.

The exclusion criteria included articles that do not evaluate factors that can affect the magnetic attractive force; articles that do not measure the magnetic attractive force; types of articles other than in vitro; and articles that measure retentive force on attachments instead of dental magnetic attachments.

### 2.3. Data Collection and Analysis

Two reviewers (Kusumadewi and Risdiana) extracted all the data. The data collected from the reviewed paper are presented in a table of research characteristic by categorizing information that is similar to make it easier to identify and analyze research variables. In studies where retentive force of magnetic attachments were evaluated and compared to other types of attachments (such as the locator and ball attachment), only data that correspond to the interests of this literature review will be extracted. All data that have been collected are then discussed with all authors to reach an agreement. The data then are classified into groups of factors that can affect the magnetic force.

### 2.4. Assessment of Risk of Bias

The assessment of risk of bias was performed by two authors (Damayanti and Rukiah). The risk of bias evaluation was modified from a previous in vitro study [11–13]. It was evaluated according to the article's description of the following parameters for the study's quality assessment: factory-made standard magnets, attractive forced measurement, and treatment of research based on a standardized method (For example, such as ISO or previous studies), sample size descriptions, blinding of the operator of the testing machine, and testing procedures performed by the same operator. If the author reported parameters in the article, it is noted Yes (Y), and if not reported, then it is noted No (N). The articles reporting 5–6 items were classified as a low risk of bias, 3–4 as medium risk and 1–2 as high risk.

## 3. Result

**Figure 1:**
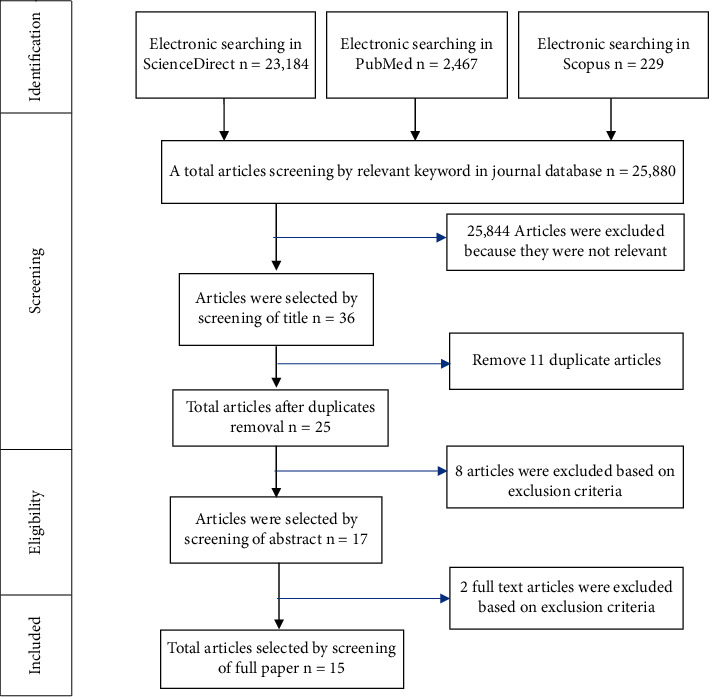
Selection procedures according to the PRISMA guidelines.

A total of 25,880 articles were obtained during a search on three journal databases: PubMed (2,647), ScienceDirect (23,184), and Scopus (229). The last search was done on 11 May, 2022. Thirty-six articles that meet the inclusion criteria were selected through title screening, and 11 duplicate articles were removed. After analyzing the abstract, 8 articles were excluded based on exclusion criteria. One article only evaluated corrosion, one article was in vivo research, one article was in silico research, two articles did not evaluate factors that affected the magnetic attraction force, one article did not measure the magnetic attraction force, one article evaluated the retention force in ball attachments, and one article only measured magnet flux density. There were 17 articles analyzed through full-text articles, and two articles were excluded because both of the articles did not measure the magnetic attraction force and did not evaluate factors that affected the magnetic attraction force. The remaining 15 articles are then reviewed for data extraction. Selection procedures according to the PRISMA guidelines in this literature review are shown in Figure 1.

Table 1 shows the summary of characteristics of each study reviewed in this literature review. The type of magnets, measurement methods, and research treatments vary in each article. Factors that can affect the magnetic force were classified into groups as seen in Table 2. The most studied factors are temperature (4 articles) and insertion-removal cycle (4 articles), followed by corrosion (3 articles).

### 3.1. Risk of Bias

Of the 15 studies included, 9 studies presented a medium risk of bias, and 6 studies showed a high risk of bias. None of the articles had a low risk of bias. The results are presented in Table 3.

## 4. Discussion

Many factors can influence the magnitude of the magnetic attractive force. The temperature has been proven in research to reduce the magnitude of the attractive force. Magnets consist of a group of magnetic atoms. Each magnetic atom has an electron structure, each magnetic electron has an electric charge, and it moves around its orbit. The movement of these electrons produces a magnetic field around the atom. There are two kinds of electrons, namely, paired electrons and unpaired electrons. Paired electrons are in the inner orbit close to the nucleus of the atom; these electrons do not spin around their orbits. The unpaired electrons are in the outer orbit of the atom, spinning around its orbit. The rotation of these electrons produces a magnetic field. Atoms have a magnetic field in an orderly arrangement when certain materials are magnetized, and when the material is demagnetized, the magnetic fields are arranged randomly. High temperatures interfere with the activity of electron rotation in its outer orbit [4]. The magnetic force decreases when exposed to high temperatures exceeding the Curie temperature point. The demagnetization process begins at temperatures over 200°C. NFeB will lose its magnetic force at temperatures over 400°C. This happens because when exposed to high temperatures, the orientation of the magnetic atoms becomes disturbed [4].

Heating the keeper at high temperatures has also been shown to reduce magnetic attractive forces. Keeper heating occurs in the process of attaching the keeper to the cast post by casting bonded and welding bonded. Keeper heating causes changes in keeper morphology such as surface flatness [27], changes in shape and size [15], as well as increasing the surface roughness [14, 15]. The greatest retention of the magnetic attachment is obtained when the magnet and the keeper are completely adhered plane to plane. Roughness of the keeper surface, a less flat surface, will reduce the close contact with the magnetic assembly. If there are any gaps between the magnet and the keeper, the attractive force will noticeably decrease [27]. Therefore, a polishing process is required on the keeper after casting that must be done carefully to restore the magnetic force [15].

Heating the keeper also causes corrosion to the keeper, which consequently reduces the magnetic attractive force [28, 29]. Casting bonded is a technique that is often used in daily practice to attach a keeper to a cast post; however, in some studies, casting bonded is the technique that most influences magnetic attraction compared to direct bonded and welding bonded techniques [14, 15]. The direct bonded keeper on a cast post by means of cementing is recommended because in this technique, the keeper does not go through the heating process so that the original shape of the keeper can be maintained [15, 27]. In addition to the high temperature in the casting process, a study by Boeckler et al. [17] has shown that sterilization of the keeper using an autoclave at a temperature of 134°C for 10 minutes resulted in a reduction in magnetic attraction although, it was not statistically significant, and it was suggested that magnetic implant abutments should be sterilized with caution to reduce the risk of alterations of the retention properties [17].

Corrosion has been shown in research to reduce magnetic attraction [5, 9]. Research by Boeckler et al. [10] shows that the magnetic assembly or keeper that has been encapsulated with an anticorrosion casing can experience corrosion. Magnets and keepers immersed in corrosive liquids dissolve metal ions, and this is the beginning of the corrosion process [10]^.^ Other studies have also shown that overdenture retention failure in patients results from corrosion of magnets. Chung et al. [30] in their study found that corrosion was the cause of failure of magnetic attachment retention in the overdenture after 34 months of clinical use. The dental magnets used are composed of NdFeB alloy, which employ an open circuit, and it was a disc-shaped magnet completely encapsulated by a stainless plate. The corrosion is caused by damage to the encapsulating material and failure of welding. Corrosion begins at the surface of the encapsulating material which is subjected to wear and at the welded zone. This is followed by a breakdown of the magnetic material. The rate of corrosion increases after the magnetic material is broken down [30]. Dental magnetic attachments that have been damaged and are in a corrosive environment will experience corrosion. Corrosion to magnets will continue over time; the magnetic attractive force will gradually decrease, until about 12 months later, it will disappear, where at that point, there is no magnetic attractive force remaining [4].

Magnets are susceptible to corrosion, especially when in an environment containing chlorides. The magnetic attachment in the oral cavity will always be in contact with saliva. Saliva in the oral cavity contains chloride [3]. The potential for corrosion increases under conditions of decreased salivary pH and increased chloride concentrations in saliva [31]. Mechanism of corrosion in magnets can occur due to damage of the metal casing or due to the diffusion of ions or liquids through an epoxy seal [9]. Damage to the metal casing causes the magnet to be exposed, and direct contact between the magnet and saliva will result in magnet corrosion. Corrosion or physical damage to magnets results in changes in the magnetic chemical composition. This can cause the magnet to lose its magnetic properties [4, 7].

The durability of the overdenture and corrosion in dental magnetic attachments is also influenced by the patient's ability to maintain dentures. Patients have to be educated properly to ensure the hygiene of oral and overdenture. Plaque accumulation and poor oral hygiene can interfere with oral health and can affect the function of the magnetic attachment. Magnetic corrosion is reported to increase due to the presence of biofilms produced by oral microflora [4].

A dentist has to provide oral hygiene instructions to patients for daily maintenance of dentures. Dentures with the magnetic assembly must be removed during sleep; in addition to tissue health, this aims to reduce the contact time between the assembly of the denture and saliva. Dental magnetic attachments are not permitted to be cleaned using tools or materials that are corrosive or abrasive that will scratch or damage the metal casing material. Cleaning agents containing chlorides should be avoided for stainless steel materials [32].

Magnetic attractive force can be influenced by insertion-removal cycles. Of the 4 studies that evaluated the insertion-removal cycle [21, 22, 24, 25], only one study [22] stated that this cycle can significantly decrease the magnetic force. This study was conducted on NdFeB magnet, and significant changes in the magnetic force began to occur after the insertion-removal cycle 5000 times or equivalent to 2.3 years of clinical use. It is recommended that magnetic attachments be replaced periodically after a long period of use [22].

The magnetic attractive force can also be influenced by clinical procedures. Installation of the magnetic assembly in a denture base must be carried out with appropriate clinical techniques and procedures. The attractive surface of the magnetic attachment must not be grinded because it will damage the protective casing and cause the magnet to be susceptible to corrosion [8]. The dentist should be able to determine the selection of the right type of magnet for successful treatment of the magnetic overdenture. Closed field magnets and NdFeB magnets produce significantly greater attractive forces than open field or SmCo magnets [18]. This can be taken into consideration in choosing the right magnet for the patient.

This study has several limitations. The results of this review should be interpreted with caution because the in vitro studies have some limitations when trying to simulate in vivo conditions. Although some studies have simulated the condition of the oral cavity, but the condition of the oral cavity is much more complex and difficult to replicate in laboratory studies. There are no studies with a low risk of bias, and the majority of studies have a medium risk of bias. Even so, this in vitro literature study can provide an overview of factors that can affect the magnetic attractive force.

## 5. Conclusion

Many factors can affect the magnetic attraction force of the dental magnetic attachment. Among the 11 factors reviewed in this paper, corrosion is the most likely factor to occur because the dental magnetic attachment is always in the oral environment, which contains corrosive saliva and is susceptible to damage due to mastication forces. The factors that can affect the magnetic force are needed to be known as a guide for dentists in choosing the type of magnetic attachment, performing the right clinical technique in the treatment of the magnetic overdenture, and delivering the right information to the patient for maintenance of the magnetic overdenture.

## Figures and Tables

**Table 1:** Summary of characteristics of each study.

No.	Author, year	Type of magnets	Type of attractive force gauge; cross head speed; measurement of each sample	Treatment in research	Main outcomes
1	Yiu et al. 2004 [5]	Nonencapsulated: (i) NdFeB (ii) Prototype iron-platinum (FePt)	Instron testing machine 2 mm/min of speed 3 times of measurement	Immersion in 3 media, namely, 1% lactic acid solution (pH 2.7), 0.1% sodium sulphide solution (pH 12), and adjusted artificial saliva (pH 6.8) were compared after 28-day and 60-day periods.	NdFeB magnet has poor corrosion resistance in artificial saliva, 1% lactic acid, and 0.1% sodium sulphide. FePt magnet has improved corrosion resistance compared to NdFeB in both artificial saliva and 1% lactic acid.

2	Chao et al.2005 [14]	Keepers (Magnedisc 800)	Universal testing machine (AG-10TA; Shimadzu) 5 mm/min of speed 6 times of measurement	3 groups of treatment: casted dowel keeper, laser-welded dowel-keeper, and control group. The alloy used is Ni-Cr.	Vertical magnetic retentive force of the control group is higher (5.6 ± 0.3 N) than the laser welded (4.2 ± 0.2 N) and casted dowel keeper groups (3.8 ± 0.3 N). Vertical magnetic retentive force of the laser-welded dowel-keepers was significantly higher than the casted dowel keeper group. Laser welding had less influence on the magnetic retentive forces than casting.

3	Ohashi et al. 2007 [15]	NdFeB: (i) Hicorex slim 3013 cup yoke type (ii) Magfit EX400 Sandwich yoke type	Digital force gauge (FGC-1) (Speed and measurement were not mentioned)	6 groups of treatment (Hicorex and Magfit): Untreatment/control group, heated, cast bonded with Ag-Pd alloy, cast bonded with Ag-Pd alloy and polished, cast bonded with type 3 gold alloy, and cast bonded with type 3 gold alloy and polished.	The attractive force of the Hicorex system was reduced by cast bonding. Attractive force of the Magfit system was reduced by both heating and cast bonding. Attractive force of both systems was recovered through the polishing process.

4	Huang et al. 2008 [16]	NdFeB: Magfit DX400	YS-31D dial tension gauge 10 times of measurement Speed was not mentioned	Repeated gliding motion over a 5 mm distance was applied on each specimen until 30,000, 50,000, or 90,000 cycles.	Retentive force of the magnet did not change significantly after 90000 gliding cycles.

5	Boeckler et al. 2009 [17]	NdFeB, closed field, mono system: (i) Magfit dome type (ii) Magfit flat type (iii) Magna cap NdFeB, open field, mono system: (iv) WR Magnet SmCo, Open field, duo system: (v) Titan magnetics	Universal test machine (Z005) 20 mm/min of speed 10 times of measurement	All magnets were sterilized for 10 minutes at 134°C in a dental steam autoclave.	Autoclave sterilization caused a nonsignificant reduction in the magnetic attractive force of 0.04–14.6%.

6	Akin et al. 2011 [18]	NdFeB, closed field: Hilop, Hicorex NdFeB, Open field: Dyna SmCo, Open field: Steco	Universal testing machine (Lloyd LF Plus) 50 mm/min of speed 10 times of measurement	All of magnetic attachments were measured in a universal testing machine.	NdFeB and closed field magnets produce significantly greater attractive forces than SmCo or open field magnets. The strongest attractive force was found with the Hilop system (9.2 N), and the lowest force was found with the Steco system (2.3 N).

7	Hasegawa et al. 2011 [19]	Gigauss D400, D600, D800, D1000 NdFeB magnets Closed magnetic circuit	Universal testing machine (EZ-Test, Shimadzu) 5 mm/min of speed 5 times of measurement	Measuring magnetic force on a combination of 6 different sizes of the keeper and assemblies: The D400 keeper was tested in combination with D400, D600, and D800 assemblies. The D600 keeper was tested in combination with D600, D800, and D1000 assemblies.	The retentive force was the highest when the same-sized magnetic assembly and keeper were used. The larger the size difference between the keeper and the magnetic assembly, the greater the decrease in the retentive force.

8	Yang et al. 2011 [20]	Magfit flat type and Magfit SX2	Universal testing machine (SV-52 NA) 1 mm/sec of speed 10 times of measurement	Implant inclination: 0°, 15°,30°, 45°	The retentive force decreases with an increase in implant inclination, but the changes in the retentive force were minimal.

9	Chung et al. 2011 [21]	NdFeB: (i) Magnedisc 800 (ii) Neomagnet	Instron 50 mm/min of speed Measurement was not mentioned	Repeated insertion and removal of the overdenture for 5400 cycles and the cyclic loading test (0–78 N) for 100,000 loading cycles.	No significant changes in the retentive force after repeated dislodging or cyclic loading.

10	Akin et al. 2013 [9]	NdFeB, closed field: Hilop, Hicorex NdFeB, Open field: Dyna SmCo, Open field: Steco	Universal testing machine (Lloyd LF Plus) 50 mm/min of speed 10 times of measurement	3 groups of treatment: (i) magnetic attachments were immersed in lactic acid 1% pH 2.3 and NaCl 0,9% pH 7,3. (ii) magnetic attachments were put through 10,000 thermal cycles (5°C/55°C).	Magnetic attachments showed lower attractive force after immersion in corrosive environments. Closed-field systems were not affected by the thermocycling procedures and were more resistant than open-field systems.

11	Hao et al. 2014 [22]	Closed field system Magfit EX 600 W	Universal testing machine (Instron) 5 mm/min of speed 5 times of measurement	Measuring the retentive force after 5000, 10,000, and 20,000 insertion and removal cycles (vertical direction).	The initial maximum retentive force of Magfit EX 600 W was 3.3 N. The mean retentive force decreased significantly after 5000 (2.7 N), 10 000 (2.1 N), and 20 000 (1.9 N) insertion-removal cycles.

12	Lee et al. 2017 [23]	Closed field system DX 600 SX-L	Instron testing machine 50 mm/min of speed 10 times of measurement	Experimental groups were designed by number (2 and 4 implants) and the type of magnetic attachment (flat and cushion type). Three directions of tensile force: vertical, oblique, and anterior-posterior were applied to measure the retentive force.	The more implant placed, the greater retentive force obtained, regardless of the type of the magnetic attachment. In all groups, the anterior-posterior retentive force is the lowest among 3 different directions of the dislodging force. The flat type of the magnetic attachment is more retentive than the cushion type of the magnetic attachment when oblique direction of the dislodging force is applied.

13	Kang et al. 2019 [24] (Materials, 2019; 12:1–12)	NdFeB: Magfit SX-L	Universal testing machine (Instron) 5 mm/min of speed 5 times of measurement	(i) Insertion-removal cycles in an artificial oral environment (standard artificial saliva at (37° ± 2°) C, a cyclic rate of 20 cycle/min. (ii) Measurement of retention at 0, 750, 1500, and 2250 cycles.	Average loss in retention 3,38% (0–2250 cycles). No significant differences in the retentive forces of the magnetic attachments before and after insertion-removal cycles.

14	Kang et al. 2019 [25] (J Magn, 2019; 24 : 733–8)	Magden	Universal Tester (i) Instron 5900: 5 mm/min of speed 50 mm/min of speed (ii) Instron 5940: 3 mm/min of speed 5 times of measurement	3 groups of treatments: Measure the retentive force (i) at a different size of assembly diameter. (ii) at a different crosshead speed (5 and 50 mm/min). (iii) at 1500 cycles of repeated detachments (immersed in artificial saliva 37° ± 2°C).	(i) The retentive force increases as the diameter of the magnetic attachment increases and decreases as the crosshead speed increases. (ii) The retentive force increases after 1500 detachment cycles.

15	Kusumadewi et al. 2021 [26]	NdFeB, stainless steel encapsulated: Magfit DX 600	Universal Testing Machine (Llyod LRX-Plus 5 kN) 50 mm/min of speed 10 times of measurement	4 groups of treatment: Magnetic attachments were immersed in acid solutions with a pH of 3.8 and 5.8 in 7 and 14 days of immersions.	Immersion of magnetic attachments in both acidic solutions and time of immersions caused surface corrosion, reduces magnetic attraction, and results in dissolution of Fe ions. The highest reduction in the magnetic force (25.15%) occurred at a pH of 3.8 with time of immersion of 14 days.

**Table 2:** Factors that affected attractive magnetic force based on reviewed articles.

No	Factors affected attractive force	Study	Year
1	Temperature	Chao [14]	2005
		Ohashi [15]	2007
		Boeckler [17]	2009
		Akin [9]	2013

2	Corrosion	Yiu [5]	2004
		Akin [9]	2013
		Kusumadewi [26]	2021

3	Keeper surface morphology	Ohashi [15]	2007

4	Type of magnet (circuit system, alloy, and shape)	Akin [18]	2011
		Lee [23]	2017

5	Keeper-assembly size	Hasegawa [19]	2011
		Kang (J Magn, 2019; 24 : 733–8) [25]	2019

6	Inclination	Yang [20]	2011

7	Insertion-removal cycle	Chung [21]	2011
		Hao [22]	2014
		Kang (J Magn, 2019; 24 : 733–8) [25]	2019
		Kang (Materials, 2019; 12:1–12) [24]	2019

8	Gliding/loading cycle	Huang [16]	2008
		Chung [21]	2011

9	Number of magnets	Lee [23]	2017

10	Crosshead speed	Kang (J Magn, 2019; 24 : 733–8) [25]	2019

11	Force direction	Lee [23]	2017

**Table 3:** Risk of bias considering parameters reported in reviewed articles.

No.	Study	Standardized magnet	Standardized treatment	Standardized measurement	Sample size descriptions	Blinding of the operator	One operator	Risk 1–2 = H; 3–4 = M; 5–6 = L
1	Yiu et al. 2004 [5]	Y	Y	N	Y	N	N	M
2	Chao et al. 2005 [14]	Y	N	N	Y	N	N	H
3	Ohashi et al. 2007 [15]	Y	N	N	Y	N	N	H
4	Huang, 2008 [16]	Y	N	N	Y	N	N	H
5	Boeckler et al. 2009 [17]	Y	Y	Y	Y	N	N	M
6	Akin et al. 2011 [18]	Y	N	Y	Y	N	N	M
7	Hasegawa et al. 2011 [19]	Y	N	N	Y	N	N	H
8	Yang et al. 2011 [20]	Y	Y	Y	N	N	N	M
9	Chung, 2011 [21]	Y	Y	N	Y	N	N	M
10	Akin et al. 2013 [9]	Y	Y	Y	Y	N	N	M
11	Hao et al. 2014 [22]	Y	N	N	Y	N	N	H
12	Lee et al., 2017 [23]	Y	N	Y	Y	N	N	M
13	Kang et al. 2019 (Materials, 2019; 12:1–12) [24]	Y	Y	Y	Y	N	N	M
14	Kang et al. 2019 (J Magn, 2019; 24 : 733–8) [25]	Y	Y	Y	Y	N	N	M
15	Kusumadewi et al. 2021 [26]	Y	N	N	Y	N	N	H
Note:	*Y* = reported in the article							
*N* = Not reported	*H* = High risk	*M* = Medium risk	*L* = Low risk					
